# Applying the trans‐contextual model to promote sport injury prevention behaviors among secondary school students

**DOI:** 10.1111/sms.14002

**Published:** 2021-07-06

**Authors:** Alfred S. Y. Lee, Martyn Standage, Martin S. Hagger, Derwin K. C. Chan

**Affiliations:** ^1^ Centre for Child and Family Science Faculty of Education and Human Development The Education University of Hong Kong Hong Kong; ^2^ School of Public Health The University of Hong Kong Hong Kong; ^3^ Centre for Motivation and Health Behaviour Change Department for Health University of Bath Bath UK; ^4^ SHARPP Lab Psychological Sciences University of California Merced CA USA; ^5^ Faculty of Sport and Health Sciences University of Jyväskylä Jyväskylä Finland; ^6^ School of Psychology Curtin University Perth WA Australia

**Keywords:** digital health intervention, mobile health, self‐determination theory, sport injury, theory of planned behavior

## Abstract

The current study tested the effects of an intervention based on the trans‐contextual model (TCM) on secondary school PE students’ sport injury prevention behavior and on theory‐based motivational and social cognition mediators. Participants were PE students (*N* = 1168; *M*_age_ = 13.322 ± 1.045, range = 12–16; female = 51.721%) who participated in a 3‐month cluster‐randomized controlled trial. Schools were randomly assigned to a treatment group, in which PE teachers received training to be more supportive of psychological needs in teaching sport injury prevention, or a control group, in which PE teachers received no training. Participants completed survey measures of TCM variables and self‐reported sport injury prevention behavior at baseline and at 3‐month post‐intervention follow‐up. The proposed TCM model exhibited adequate fit with the data, χ^2^ = 143.080 (df = 19), CFI = 0.956, TLI = 0.916, RMSEA = 0.078 (90% CI = 0.066–0.090), and SRMR = 0.058. We found positive, statistically significant direct intervention effects on changes in perceived psychological need support (β = 0.064, *p* = 0.020). We also found positive, significant direct (β = 0.086–0.599, *p* < 0.001) and indirect (β = 0.002–0.027, *p* = 0.020–0.032) intervention effects on changes in TCM variables and behaviors to prevent sport injuries. Our findings support the TCM as a useful framework for building an intervention for promoting sport injury prevention behaviors among secondary school students.

## 1 INTRODUCTION

Sport injury is the top reason for seeking medical attention among youth in many regions, including North America,^1^ Australia,^2^ and European Union.^3^ Adopting sport injury preventive behaviors, such as carrying out a warm‐up before sport participation, completing cool‐down exercises after sport,^4^ conducting regular muscle strengthening exercises,^5^ and performing sports with proper technique and equipment,^6^, ^7^ has been identified as effective ways to minimize acute and chronic sport injuries.^8^ Failure to adhere to these injury prevention strategies may heighten the risk of sport injury.^9^ Therefore, researchers in the behavioral sciences have employed social psychological theories to develop an understanding of the factors that are related to engagement in sport injury preventive behaviors with the ultimate goal to inform efficacious interventions to reduce the rate of sport injuries.^10^, ^11^ The promotion of sport injury prevention is important not only in competitive sports, but also in school and during leisure‐time physical activity. Students may learn and adopt sport injury prevention guidelines during physical education (PE) lessons,^12^ and sport safety education is typically included in the PE curriculum.^13^ PE teachers, therefore, have an important role to play in educating young people in sport injury prevention.^12^ Yet, the application and maintenance of sport injury prevention behaviors may not occur in unsupervised sport contexts outside of the school environment (ie, unstructured and leisure‐time physical activities).^12^, ^14^ Rather, PE teachers may contribute to facilitating students’ motivation to engage in sport injury preventive behaviors in out‐of‐school contexts.^12^, ^15^ In the current study, we developed and tested the effectiveness of an intervention based on an integrated social cognition and motivational model, the trans‐contextual model (TCM),^16^, ^17^, ^18^ to improve sports injury prevention behaviors in out‐of‐school contexts among secondary school students.

### 1.1 The trans‐contextual model

The TCM is a multi‐theory model that integrates constructs and hypotheses from self‐determination theory (SDT),^19^ the theory of planned behavior (TPB),^20^ and the hierarchical model of intrinsic and extrinsic motivation (HMM)^21^ into a unified model aimed at predicting motivation and behavior across multiple contexts.^16^ The original application of the TCM was in the context of predicting children's physical activity behavior in PE and out‐of‐school contexts.^16^ The TCM has also been applied to predict injury prevention and rehabilitation behaviors across contexts in the domain of sport.^12^, ^22^, ^23^, ^24^, ^25^ The model may provide a theoretical basis for informing the motivational strategies that PE teachers can apply in the classroom to facilitate students’ participation in sport injury prevention in out‐of‐school contexts.

There are three premises of the TCM: (1) Students’ perceived psychological need support from social agents is positively related to their autonomous motivation toward activities and behaviors within an educational context; (2) autonomous motivation in the educational context is positively related to autonomous motivation toward similar activities or behaviors in an out‐of‐school context; and (3) social cognition variables (ie, attitude, subjective norm, and perceived behavioral control) that underpin future participation in the behavior mediate the effects of autonomous motivation on intentions to perform, and actual participation in, activities or behavior in the out‐of‐school context.^18^, ^26^

### 1.2 Psychological need support

Based on SDT,^27^ the TCM makes the distinction between two fundamental forms of motivation that underpin behavior: autonomous and controlled motivation.^18^ Behaviors are autonomously motivated when they are performed for intrinsic (acting out of fun and enjoyment), integrated (acting consistent with life goals and true sense‐of‐self), or identified (acting to obtain self‐endorsed and valued outcomes) reasons. On the other hand, actions are viewed as being engaged in for controlled motivation when they are performed for introjected (acting to avoid guilt and shame) and externally regulated (acting for contingent reward and to avoid punishment) reasons.^28^, ^29^ Autonomous motivation is the most adaptive form of motivation, which and the form of motivation that tend to be related to behavioral persistence. This is because autonomously motivated actions are those that are most likely to satisfy basic psychological needs. Individuals who are autonomously motivated are more likely to perceive ownership of the behaviors and endorse their actions.^29^ Acting for autonomous reasons also leads to adaptive outcomes, including optimal overall functioning and positive affect.^29^ In contrast, acting out of controlled motivation means actions are performed for externally referenced reasons (eg, rewards, punishments, obligations).^28^ When behavior is regulated by controlled motives, it may lead to persistence only as long as the controlling contingencies are present and, as a result, is often not associated with long‐term persistence in contexts where the onus is on individuals to self‐regulate. Adopting behaviors for controlled reasons can often lead to maladaptive outcomes like negative affect and ill‐being.^23^, ^24^, ^30^ In the context of sport injury prevention, students’ autonomous motivation is positively associated with the sport injury prevention intention and behavior.^15^, ^31^

According to SDT,^32^ autonomous motivation can be fostered by satisfying the three innate psychological needs for autonomy (the need to experience choice and feel action emanate from the self), competence (the need to experience effectance in one’ own actions), and relatedness (the need to feel connected and cared for by significant others). It is proposed that the three needs can be satisfied by need supportive behaviors performed by significant others’ in responsible or leadership positions in the actor's immediate environment (eg, PE teachers). Need supportive behaviors include providing choices, explaining the rationale for a particular behavior, encouragement and actively listening to one's concern and accepting one's ability.^32^, ^33^, ^34^ Furthermore, individuals’ perceived need support, that is, the extent to which individuals feel that significant others support their psychological needs, is an important factor determining autonomous motivation. The positive association between perceived need support from significant others and autonomous motivation has been consistently reported in previous studies.^15^, ^26^, ^30^, ^35^, ^36^ Based on this tenet, PE teachers who teach sport injury prevention in a psychological need supportive manner would be expected to facilitate students’ perceived need support and autonomous motivation toward sport injury prevention behavior.^12^ This prediction is consistent with the first premise of the TCM.

### 1.3 Transfer of motivation

Another key premise of the TCM posits that an individual's autonomous motivation toward behaviors in one context (eg, sport injury prevention in school) is transferable to motivation toward similar behaviors performed in another, related context (eg, sport injury prevention outside of school).^18^, ^21^, ^27^ This link is based on a prediction of the HMM suggesting that experiencing autonomous motivation toward behaviors performed in one context level is likely to be positively related to autonomous motivation toward behaviors in similar contexts.^21^ The explanation for this prediction is that individuals develop motivational scripts or schemas when they experience autonomous motivation in a behavior (eg, sport injury prevention) in one context (eg, PE lesson), and the scripts are activated when cues related to that behavior are presented in another context (eg, out‐of‐school).^18^, ^21^, ^24^, ^25^, ^37^ This prediction forms the basis of the trans‐contextual effect and the second premise of the TCM.

### 1.4 Social cognition beliefs

The final proposition of the TCM predicts that autonomous motivation with respect to performing a behavior in an out‐of‐school context predicts subsequent performance of those activities mediated by the belief‐based determinants of the behavior. This prediction is based on an integration of the tenets of SDT^19^ and TPB.^20^ The mechanism behind this proposition is that individuals tend to align their beliefs about performing future behaviors with their motives. This alignment is strategic because it enables individuals to organize their beliefs and intentions with respect to performing the behavior in future. If their motivation toward the behavior in question is autonomous and the behavior is viewed as need satisfying, then their beliefs are likely aligned toward performing the behavior in future, facilitating performance of the need satisfying behavior. In the TCM, the beliefs are represented by the social cognition beliefs from the TPB: attitudes (individuals’ instrumental and affective evaluation of the behavior), subjective norms (individuals’ perceptions that significant others want them to perform the behavior), and perceived behavioral control (PBC; individuals’ perceived capability to perform the behavior).^18^, ^22^, ^38^, ^39^ The effect of autonomous motivation on behavioral intention (ie, one's willingness and planning to perform the behavior in the future) is mediated by the social cognition beliefs. For example, students with autonomous motives toward specific behaviors would be likely to report attitudes, subjective norms and PBC consistent with performing the behaviors, and form intentions to perform the behaviors in future.^30^, ^40^, ^41^, ^42^ Finally, consistent with the TPB, intentions are the most proximal determinant of behaviors and mediate effects the beliefs on behaviors. Research, including a recent panel study in sport injury prevention, supported these predictions and the temporal ordering of the proposed effects.^43^ Some theorists argue that subjective norms may reflect perceived social pressure which should be aligned closer with controlled motivation than autonomous motivation.^18^, ^44^ However, studies have demonstrated that subjective norms positively correlated with both controlled and autonomous forms of motivation.^37^, ^38^, ^45^, ^46^ Overall, autonomous motivation is a more consistent positive predictor of social cognition beliefs, intentions, and behaviors than controlled motivation, so building an intervention that fosters autonomous motivation by satisfying the basic psychological needs might serve as a potential solution for promoting one's attitude, subjective norms, and PBC of the given behavior.

### 1.5 Evidence for the TCM

Research using the TCM as the framework for health promotion has been growing over the past decade and the model has been applied in multiple contexts, including physical activity,^16^ anti‐doping in sport,^37^ injury rehabilitation,^24^ academic performance,^22^, ^23^ and sport injury prevention.^39^ Findings from this research have consistently supported the premises of the model including links between perceived need support and autonomous motivation in the initial context (eg, education, school), trans‐contextual relations between autonomous motivation (eg, across educational and out‐of‐school contexts), and indirect relations between autonomous motivation in the related context (eg, out‐of‐school, leisure‐time) and behaviors performed in that context mediated by beliefs and intentions from the TPB. Research has also supported invariance in the relations across studies and national groups.^26^ However, to date, few studies have used the model as a basis for intervention or demonstrated how changing key constructs in the model (eg, perceived need support, autonomous motivation) affects change in key outcomes (eg, intentions and behaviors). Such research will also provide more robust evidence for the proposed ordering of constructs in the model and potential causal effects, which cannot be ascertained from correlational data.

### 1.6 The present study

In the current study, we developed and tested the efficacy of a theory‐driven intervention based on the TCM. The intervention aimed to promote secondary school students’ autonomous motivation and intentions toward, and actual participation in, sport injury prevention behavior via the provision of psychological need support training of PE teachers. The intervention consisted of a face‐to‐face workshop and a theory‐driven smartphone application to promote psychological need supportive style of PE teachers in teaching sport injury prevention among secondary school students. We hypothesized that the intervention would facilitate students’ autonomous motivation and social cognition pattern of sport injury prevention according to the psychological mechanism proposed in the TCM.^18^, ^23^ Based on the TCM,^18^ we proposed the following hypotheses:

H1: The intervention would have a positive and significant direct effect on students’ perceptions of psychological need support for sport injury prevention offered by their PE teachers;

H2: The intervention would have positive and significant indirect effects on changes in students’ in‐school autonomous motivation, and out‐of‐school autonomous motivation, attitude, subjective norm, PBC, intention and behavior regarding sport injury prevention.

## 2 METHOD

### 2.1 Participants and recruitment procedures

We sent out invitations to 462 secondary schools in Hong Kong inviting them to participate in the study. Three schools and their PE teachers (*N* = 6) agreed to participate in the study. Nonparticipating schools (*N* = 0359) either did not respond or declined the invitation due to a busy schedule and for logistical reasons. Lower form secondary school students (Secondary 1 to Secondary 3) from participating schools were eligible to take part in the study. We focused on this age group because it is the beginning stage of secondary school education where sport safety is especially important for reducing the risk of sport injury in the later stages of physical education.^14^, ^47^ Invitation and informed consent forms were sent to the students and their parents. Finally, 1168 junior (Secondary 1 to Secondary 3, equivalent to 7th to 9th grade in the US school system) secondary school students (*M*_age_ = 13.322 ± 1.045, range = 12–16; female = 51.721%) and their parents signed the informed consent form and agreed to take part in the study. The student participants had to attend two compulsory PE lessons per teaching week. According to baseline assessments, 74.882% of participants took part in sports or physical activities outside of school. Specifically, they spent 3.561 (SD = 2.908) hours on sport or physical activities every week and experienced 1.268 (SD = 3.376) sport injuries, defined as “injured during any sports” in the past 6 months on average. It is important to note that data from the control group in the current study were previously reported in a prospective study on the predictive validity used for analyzing the longitudinal relationships of the TCM over 3 months in a sport injury prevention context.^15^ The previous study was correlational in design^15^ and did not test hypotheses relating to the effects of changing the TCM variables through intervention on injury prevention behavior. Our current study makes an original contribution to knowledge because it is the first to test whether manipulating psychological need support in a sport injury prevention setting led to change in TCM variables and sport injury preventive behavior. Our findings may offer formative evidence on how the TCM can guide interventions to promote behavior change in a sport injury prevention setting in schools.^48^

### 2.2 Study design and procedure

**FIGURE 1:**
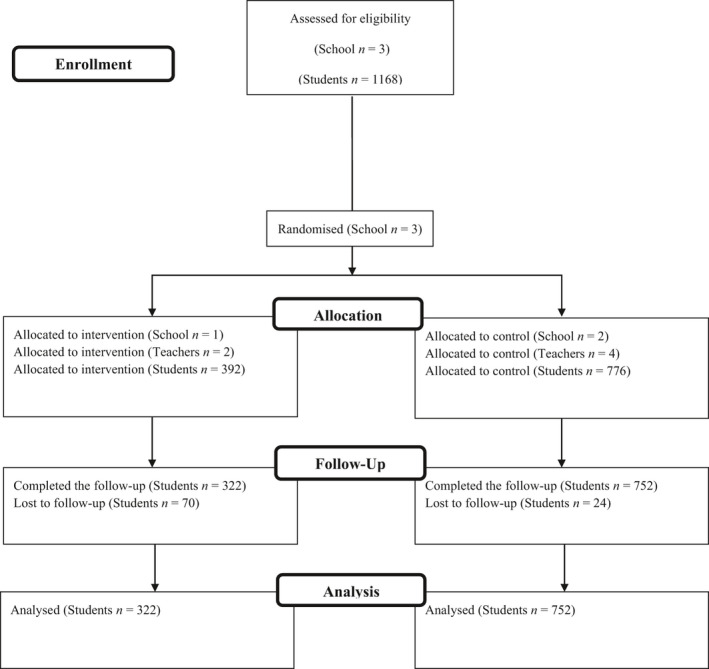
CONSORT flow diagram of the intervention driven by TCM for the promotion of sport injury prevention among secondary school students

Ethical approval was obtained from the first author's institution [approval number = EA1604014]. The current study was a 3‐month cluster‐randomized control trial with two waves of assessment (ie, at baseline pre‐intervention and at 3 months post‐intervention). Participating schools were randomly assigned to either intervention or control condition of the intervention using a computer ballot by an independent research assistant. The randomization resulted in one school being allocated to the treatment condition and two schools to the control condition. The two PE teachers from the school allocated to the treatment condition received the intervention workshop and the smartphone phone application (namely, “Sport Safety Easy”) after the baseline assessment. Student participants were asked to complete a survey package that measured the psychological variables of the TCM (eg, psychological need support, motivation, social cognition variables) at baseline and follow‐up assessment occasions. A summary of school, teacher, and participant flow through the study is presented in Figure 1.

### 2.3 Measures

#### 2.3.1 Psychological need support

We measured participants’ perception of PE teachers’ psychological need support using the sport injury prevention version of the Health Care Climate Questionnaire (HCCQ).^49^ The 6‐item Chinese version of HCCQ has been applied in sport injury prevention contexts and has reported good internal consistency and validity.^24^, ^31^ Participant responses were provided on 7‐point scales (1 = *not at all true* to 7 = *very true*).^1^

#### 2.3.2 Sport injury prevention motivation

We used 6 items from the Chinese version for the Treatment Self‐Regulation Questionnaire^50^ to measure participants’ autonomous motivation (6 items) to prevent sport injuries.^24^, ^31^, ^42^ In this study, participants were asked to complete the 6 items of the autonomous motivation scale twice, once for in‐school and once for out‐of‐school sport injury prevention. Participant responses were provided on 7‐point scales (1 = *not at all true* to 7 = *very true*).

#### 2.3.3 Social cognition variable

Items assessing the TPB constructs were developed according to standardized guidelines ^51^ with the behavior of interest “*follow all required safety procedures to reduce the likelihood or severity of injury*.” Participants completed measures of attitude (6 items), subjective norms (3 items), PBC (5 items), and intention (3 items) with responses provided on 7‐point scales (eg, 1 = *strongly disagree* to 7 = *strongly agree*). Participants completed Chinese versions of the measures previously developed for use in injury prevention contexts.^24^, ^31^

#### 2.3.4 Behavior

We used the Self‐Reported Injury Prevention Adherence Scale ^39^ to evaluate students’ sport injury prevention behavior. Participants completed the Chinese version of the scale developed in previous studies.^15^, ^39^ The scale consists of 8 items measuring participants’ effort (4 items) and frequency (4 items) of preventing sport injury (eg, following all required safety procedures, seeking safety advice from others, and avoiding re‐injury). Participant responses were provided on 7‐point scales (1 = *never*/*minimum effort* to 7 = *very often*/ *maximum effort*).

### 2.4 Intervention materials

The intervention program consisted of two components, a workshop and a smartphone application. Each aimed to promote participating PE teachers in schools assigned to the treatment group to use a psychological need supportive style in teaching sport injury prevention in their PE classes.

#### 2.4.1 Workshop

The workshop was a face‐to‐face session with the PE teachers facilitated by the first author and a trained research assistant. In the workshop, the PE teachers were provided with information on sport injury prevention, including an introduction to motivation, instruction on how to be psychological need supportive in teaching sport safety, different warm‐up protocols^52^ (eg, FIFA 11+ ^9^), and information reinforcing sport safety techniques (eg, landing techniques and cool down). The smartphone application was installed in the smartphones of PE teachers in the intervention schools, and they were given a tour on its use to support their need supportive teaching of injury prevention as part of the intervention. The workshop lasted for approximately one and half hour. The educational materials of this workshop are provided in Appendix II (https://osf.io/gw3cu/).

#### 2.4.2 Smartphone application

We developed “Sport Safety Easy,” a smartphone application based upon the concepts of the TCM and findings of previous studies on need supportive instructional styles.^12^, ^18^ The smartphone application aimed to facilitate PE teachers’ need supportive style in teaching sport injury prevention in their regular PE lessons. The smartphone application included information on the causes of sport injury, treatment of sport injury, sport injury prevention methods, and demonstration videos on sport injury prevention from the FIFA 11+ program.^52^, ^53^ The application also introduced a number of strategies and concepts related to psychological need supportive teaching style in a sport injury prevention context. The application highlighted the principles behind, and importance of, psychological need support, provided examples on how to be need supportive, gave scenarios that PE teachers might encounter when teaching sport injury prevention, and listed the pros and cons for teaching sport injury prevention. The application also provided ongoing support through daily notifications to support PE teachers. Screenshots of the smartphone application are presented in Appendix III (https://osf.io/gw3cu/).

### 2.5 Data analysis

Preliminary analyses consisted of descriptive statistics of the TCM variables and independent *t*‐tests. There were no significant differences on study variables between the intervention and control group during the baseline assessment. We conducted path analysis to examine the goodness‐of fit and parameter estimates (ie, direct and indirect effects) of a model specifying the hypothesized effects of the intervention on TCM constructs and related mediation effects. The analysis was conducted using Mplus version 7.2 with a maximum likelihood estimation method. Missing data were imputed using the full‐information maximum likelihood method.^54^ Intervention effects were tested using a dummy‐coded variable (ie, 0 = control group; 1 = treatment group) as a direct predictor of students’ perceived psychological need support (H1) and indirectly on other TCM variables (H2). In order to examine intervention effects on changes in the TCM variables and behaviors, we computed standardized residual change scores for all model variables.^55^, ^56^ Change scores were generated by regressing the post‐test measures on the baseline measures (ie, a positive value indicated an increase or positive change over time) for each variable. Such change scores have been suggested to be a more reliable score than simple difference scores.^57^ We used the conventional fit indices to assess the model fit,^58^ including the comparative fit index (CFI), Tucker‐Lewis index (TLI), root mean square error of approximation (RMSEA), and standardized root mean square residual (SRMR). Traditional cutoff criteria for CFI and TLI (ie, 0.900) and for RMSEA and SRMR (ie, 0.080) were applied to indicate acceptable fit.^58^

## 3 RESULTS

### 3.1 Preliminary analyses

Baseline characteristics and sample mean differences of intervention and control group are displayed in Table 1. Independent *t*‐tests showed there were no group differences on the demographic and psychological variables. Descriptive statistics and zero‐order correlation matrix of the residual change scores of the TCM variables are presented in Table 2 and that of the TCM variables in T0 and T1 are available online (https://osf.io/gw3cu/).

**TABLE 1:** Descriptive statistics of the study variables

	Treatment Group	Control Group	Difference	
	(*N* = 392)	(*N* = 776)	*t*	*p*
Gender				
Male	175 (44.757%)	386 (50.065%)	1.712	0.087
Female	216 (55.243%)	385 (49.935%)		
Age	13.342 (0.970)	13.312 (1.082)	−.456	0.648
Time in Sports (Minutes Per Week)	200.858 (165.720)	220.109 (178.531)	1.600	0.110
Sport Injuries (last 6 months)	1.241 (3.487)	1.282 (3.322)	0.193	0.847
T0 Psychological Need Support	4.784 (1.329)	4.651 (1.270)	−1.658	0.098
T1 Psychological Need Support	4.842 (1.295)	4.544 (1.380)	−3.309	0.001
T0 Autonomous Motivation (IS)	4.951 (1.165)	5.002 (1.051)	0.752	0.452
T1 Autonomous Motivation (IS)	4.942 (1.099)	4.949 (1.078)	0.098	0.922
T0 Autonomous Motivation (OS)	4.863 (1.173)	4.878 (1.048)	0.211	0.833
T1 Autonomous Motivation (OS)	4.857 (1.141)	4.795 (1.109)	−0.827	0.408
T0 Attitude	5.026 (1.096)	5.031 (1.032)	0.091	0.928
T1 Attitude	5.035 (1.117)	5.007 (1.078)	−.383	0.702
T0 Subjective Norms	4.640 (1.206)	4.700 (1.053)	0.876	0.381
T1 Subjective Norms	4.670 (1.405)	4.692 (1.088)	0.427	0.669
T0 PBC	4.804 (1.088)	4.810 (1.021)	0.089	0.929
T1 PBC	4.772 (1.153)	4.817 (0.999)	0.654	0.513
T0 Intention	4.531 (1.242)	4.636 (1.085)	1.491	0.136
T1 Intention	4.565 (1.196)	4.629 (1.080)	0.857	0.392
T0 Behaviors	3.974 (1.279)	4.095 (1.236)	1.559	0.119
T1 Behaviors	4.186 (1.197)	4.116 (1.226)	−.857	0.392

Note

Descriptive statistics of gender, age, time in sports, and sport injuries (last 6 months) were taken from baseline.

T0, baseline; T1, 3‐month follow‐up; IS, in‐school context; OS, out‐of‐school context.

**TABLE 2:** Descriptive statistics and zero‐order correlation matrix of the residual change scores of the TCM variables (*N* = 1168)

Variables	1	2	3	4	5	6	7	8
1. Psychological Need Support	–							
2. Autonomous Motivation (IS)	0.415***	–						
3. Autonomous Motivation (OS)	0.410***	0.635***	–					
4. Attitude	0.390***	0.411***	0.542***	–				
5. Subjective Norm	0.378***	0.469***	0.589***	0.464***	–			
6. PBC	0.357***	0.430***	0.528***	0.507***	0.715***	–		
7. Intention	0.364***	0.466***	0.585***	0.466***	0.783***	0.673***	–	
8. Behavior	0.400***	0.426***	0.516***	0.438***	0.484***	0.432***	0.497***	–
Cronbach alpha	0.938	0.838	0.868	0.890	0.822	0.879	0.915	0.890
Mean	4.695	4.985	4.873	5.029	4.680	4.808	4.600	4.055
Standard Deviation	1.291	1.090	1.090	1.054	1.106	1.043	1.141	1.251

Note

Cronbach alpha, mean, and standard deviation displayed in the table were using the baseline data.

Abbreviations: OS, in‐school context; OS, out‐of‐school context; PBC, perceived behavioral control.

***p < 0.001.

### 3.2 Intervention effects

**FIGURE 2:**
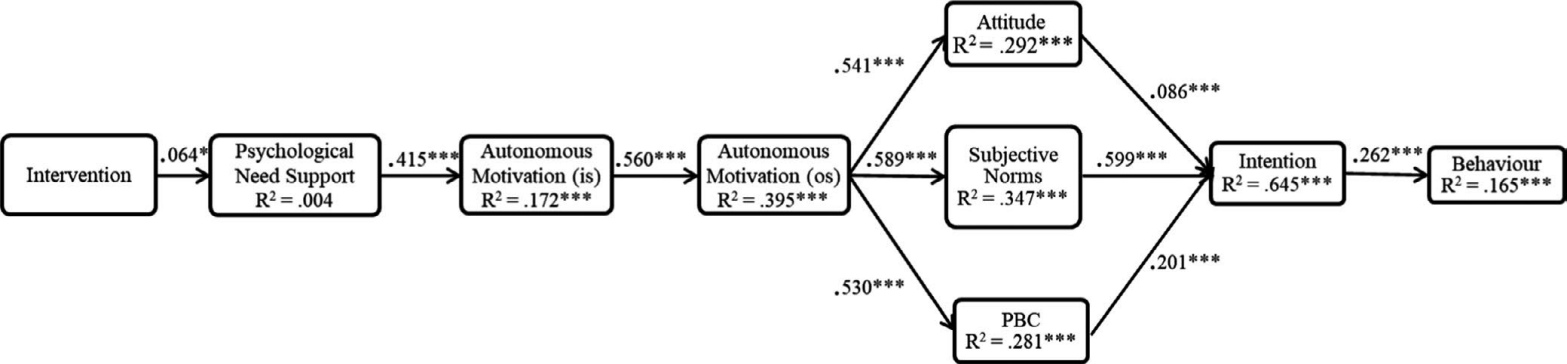
Trans‐contextual model for sport injury prevention. All path estimates were hypothesized to be positive and significant. IS = in‐school context; OS = out‐of‐school context; and PBC = perceived behavioral control. *p < 0.05 and **p < 0.01

The proposed TCM model displayed adequate fit to the data, χ^2^ = 143.080, df = 19, CFI = .956, TLI = .916, RMSEA = .078 (90% CI = 0.066–0.090), SRMR = 0.058. Standardized parameter estimates (β) for model effects and explained variance (*R* ^2^) in each dependent variable in the model are presented in Figure 2. In support of H1, the intervention had a positive and significant direct effect on the increase of PE teachers’ psychological need support (β = 0.064, *p* = 0.020). In congruence with H2, the intervention had positive and significant indirect effects on the increase in students’ in‐school autonomous motivation (β = 0.027, *p* = 0.020), out‐of‐school autonomous motivation (β = 0.015, *p* = 0.020), attitude (β = 0.008, *p* = 0.021), subjective norms (β = 0.009, *p* = 0.021), PBC (β = 0.008, *p* = 0.022), intention (β = 0.008, *p* = 0.022), and sport injury prevention behavior (β = 0.002, *p* = 0.032). Table 3 displays the parameter estimates and 95% confidence intervals of the model indirect effects. H2 was supported by all positive and statistically significant pathways within the TCM using the change scores of the TCM variables. In addition, and consistent with TCM premises, psychological need support predicted in‐school autonomous motivation (β = 0.415, *p* < 0.001). In‐school autonomous motivation predicted out‐of‐school autonomous motivation (β = 0.560, *p* < 0.001). Out‐of‐school autonomous motivation predicted attitude (β = 0.541, *p* < 0.001), subjective norms (β = 0.589, *p* < 0.001), and PBC (β = 0.530, *p* < 0.001). Attitude (β = 0.086, *p* < 0.001), subjective norms (β = 0.599, *p* < 0.001), and PBC (β = 0.201, *p* < 0.001) predicted intention and intention predicted sport injury prevention behavior (β = 0.262, *p* < 0.001).

**TABLE 3:** Results from the mediation analyses for the TCM for injury prevention

Paths	Mediator (s)	Indirect effects [95% CI]
Intervention →Auto (IS)	AS	0.027* [0.008–0.046]
Intervention →Auto (OS)	AS, Auto (IS)	0.015* [0.005–0.026]
Intervention →Attitude	AS, Auto (IS), Auto (OS)	0.008* [0.002–0.014]
Intervention →Subjective norms	AS, Auto (IS), Auto (OS)	0.009* [0.003–0.015]
Intervention →PBC	AS, Auto (IS), Auto (OS)	0.008* [0.002–0.014]
Intervention →Intention	AS, Auto (IS), Auto (OS), Intention antecedents	0.008* [0.002–0.013]
Intervention →Behavior	AS, Auto (IS), Auto (OS), Intention antecedents, Intention	0.002* [0.000–0.004]

Note

Intervention, dummy‐coded variable representing intervention effect; IS, in‐school context; OS, out‐of‐school context; AS, autonomy support; Auto, autonomous motivation; PBC, perceived behavioral control.

*p < 0.05.

## 4 DISCUSSION

The objective of the current study was to develop and test the efficacy of a TCM‐based intervention that aimed to promote sport injury prevention behaviors among secondary school students. The TCM‐based intervention consisted of a face‐to‐face workshop and a smartphone application that supported PE teachers’ use of psychological need supportive style when teaching sport injury prevention in secondary school PE lessons. The intervention was tested in a three‐month cluster‐randomized controlled design with schoolteachers and their students assigned to receive either the intervention or a no‐intervention control. A path analysis of data collected on measures of students’ TCM constructs and physical activity participation at baseline and at three‐month post‐intervention follow‐up supported the hypothesized direct and indirect effects of the intervention on change in these variables. This consistent pattern of results demonstrated that the TCM‐based intervention aimed at promoting PE teachers’ psychological need supportive style in teaching sport injury prevention was related to changes in their students’ perceived autonomy support, autonomous motivation across school and out‐of‐school contexts, social cognition beliefs, intention, and sport injury prevention behavior. Our intervention provided preliminary evidence to support the predictive validity of TCM under conditions of change, and the utility of TCM in guiding the development of an efficacious mobile health behavior change intervention in a sport injury prevention context.

Consistent with previous SDT‐based interventions, our study was effective in increasing students’ perceived psychological need support while controlling for baseline values.^49^, ^56^, ^59^ Based on meta‐analyses of need supportive interventions,^60^, ^61^ the combination of a workshop and the “Sport Safety Easy” smartphone app to promote psychological need supportive behaviors in teachers seemed to work well in promoting changes in students’ perceived psychological need support. The approach deviates from typical means to deliver SDT‐based interventions which often consist only of one‐off workshops.^62^ Yet, this design is consistent with the suggestion that such interventions would be more effective when supported by supplementary follow‐up sessions or content,^60^ and our findings seem to support this proposition. A major advantage of the use of the app alongside the workshop is that it provided ongoing support for the intervention content with daily supportive notifications and ultimately might serve to prolong and provide continuous support for the intervention.^60^ It also enabled inclusion of more varied content such as hypothetical scenarios and opportunities to demonstrate/practice need supportive styles in teaching sport injury prevention. However, we did not collect any data on PE teachers’ experience and personal opinions on the workshop content and usability of the app and is an avenue for future research. Such data will provide important information of how the content of the need supportive intervention is accepted and tolerated and how support activities such as the app may assist in assimilation and application of the intervention content.

Consistent with our hypotheses, the intervention also had positive and significant indirect effects on change in the TCM constructs. Specifically, the TCM intervention was indirectly associated with better in‐school and out‐of‐school autonomous motivation, social cognition beliefs, intention, and behavior of sport injury prevention. Findings are consistent with correlational data on TCM relations with the added advantages of demonstrating how manipulation of key constructs in the model affects change in model constructs in sport injury prevention settings.^31^, ^39^ Although the indirect effects of the intervention on the TCM variables were small, it must be stressed that effect sizes of indirect effects should not be interpreted in the same way as direct effects, given that indirect effects are multiplicative composites of a number of separate effect sizes. The key message is that the general pattern of results using an intervention and change scores were consistent with previous correlational studies that examined relations among the TCM variables.^22^, ^23^, ^24^ Current findings suggest that the TCM may have utility in promoting development of need supportive interventions and explaining their effects on behavior. Potentially, the current findings may extrapolate to developing interventions based on the TCM more broadly, for example, in representative samples of schools and also in other contexts in which the TCM has been applied such as physical activity,^17^ myopia prevention,^38^ and other educational contexts.^22^, ^63^

It is also worth noting that predicted relations among TCM model constructs were positive and statistically significant, consistent with previous studies.^15^, ^22^, ^23^, ^25^ Importantly, these effects used change scores which contrasts with the typical approach. Findings suggest that the model may be effective in modeling change in constructs over time, beyond the mere ‘static’ prediction offered in correlational studies.^18^ Results are consistent with recent research which has also provided generalized support for the efficacy of the model when accounting for temporal change in physical activity across PE and leisure‐time contexts.^64^

## 5 LIMITATIONS AND RECOMMENDATIONS FOR FUTURE RESEARCH

It is important to acknowledge some key limitations in the current study which may affect interpretation. First, our study was not randomized at the level of the participant, but at the school level, and only included three secondary schools. Within‐cluster correlations among study measures may have affected the statistical power of our study to detect effects and the estimates of intervention effects.^65^

Second, given the number of schools, it is also important to note that we cannot infer the current intervention effects will generalize to a broader sample of schools in Hong Kong or more broadly. Such generalization would only be warranted after further application of the intervention in a larger, representative sample of schools. However, the efficacy of the intervention identified in the current findings paves the way for such a broader application and provides in‐principle support for the predicted effects.

Third, we were unable to determine the extent of adoption of the intervention materials and implementation of the recommended psychological need supportive teaching strategies by teachers allocated to the treatment condition. We did not include measures evaluating the frequency of use of the smartphone application, nor did we monitor the extent to which PE teachers implement the techniques they learned in their PE classes. Future studies should account for frequency of use of the smartphone app through monitoring. For example, researchers should note how often teachers accessed the app, the total time they spent on the app, and completion of viewing the smartphone materials. They also should implement assessment of PE teachers’ adherence and motivation toward the intervention program and include means to evaluate PE teachers’ psychological need supportive teaching style prior to the intervention and at follow‐up. This would provide important fidelity and manipulation check data, essential to establish intervention efficacy.

Fourth, the current study design was unable to detect which interventional components, face‐to‐face workshops or smartphone application, yielded a larger effect on teachers’ psychological need support. Future studies are encouraged to test the independent effects of individual intervention components using different study designs (eg, factorial designs), to increase the efficiency of interventions.^66^

Fifth, our study did not include non‐self‐report measures of students’ sport injury prevention behavior. It is important to note that self‐report behavioral measures have utility and validity,^67^ and often differences in self‐report measures can be attributed to differences in reliability and mode of delivery rather than systematic bias.^68^ Nevertheless, the possibility of self‐serving bias and socially desirable responding is still a threat to study validity.^69^ For example, the use of psychometric scales to measure study variables might lead to consistency tendency in responses which artificially inflates the covariance of participants’ responses.^42^, ^70^ Therefore, we would encourage researchers to measure students’ sport injury prevention behavior using non‐self‐report measures (eg, observation) and include other indicators such as sport injury rate, severity, and types of prevention techniques adopted in future studies. Since the current intervention focused on promoting students’ sport injury prevention behaviors, sport injury outcomes (ie, sport injury rate and severity) were not recorded. We were, therefore, unable to test whether the intervention or the psychological variables of the TCM were linked to the reduction of sport injury. Yet, previous studies have provided evidence regarding the effects of psychological variables on the rates of sport injury.^10^, ^11^ Future studies should investigate whether the intervention is effective in reducing the incidence of sport injury with a long‐term follow‐up assessment.

Sixth, no treatment was provided to the control group. This design might limit the implications of the current study because it is possible that simply providing the teachers the intervention content related to preventing sports injuries may have been just as effective.^71^ However, we contend that the current study has inherent value because it demonstrates the efficacy of the intervention in populations where there is little or no alternative intervention or “usual care.” Seventh, it is also important to note that the current intervention only focused on fostering a narrow set of need supportive behaviors in PE teachers. Future interventions may be more elaborate, incorporating a greater number of motivational and change strategies aligned with SDT. In particular, future studies may explore the effectiveness of introducing other change strategies such as using non‐controlling language, provision of meaningful rationales, organization of co‐operation tasks, and avoidance of peer‐competition into theory‐driven interventions for promoting sport injury prevention among PE students.^34^, ^61^

Finally, we could not establish the casual relationship between the mediators and the outcomes because our statistical analysis may violate the “sequential ignorability” assumption.^72^ Specifically, randomization was confined to the dummy‐coded intervention variable; however, the levels of the mediators, namely psychological need support, autonomous motivation in both contexts and the social cognition beliefs, were not directly randomized. Obscure confounders may affect the links between the mediators and the outcomes, and hence, causalities were yet to demonstrate. To address this limitation, future studies are suggested to adopt comprehensive structural equation models, including the principal stratification approach and/or instrumental variable method.^61^, ^72^

### 5.1 Perspective

This study developed and tested the efficacy of an intervention that trained PE teachers to be need supportive based on the TCM in promoting secondary school PE students’ sport injury prevention behaviors. Findings support the positive effects of the intervention on PE students’ perceived psychological need support from their teachers, which was indirectly related to autonomous motivation, social cognition beliefs, and intention toward, and actual participation in, sport injury prevention behaviors. Findings may imply that supporting students’ psychological needs may not only foster better in‐school autonomous motivation, but it may also promote out‐of‐school motivation, social cognition beliefs, intention, and behavior of sport injury prevention. Results also highlight the importance of PE teachers’ psychological need support in promoting sport injury prevention behaviors. Psychological need supportive strategies, such as developing game‐like warm‐up programs, actively listening to students’ concerns, and promoting self‐comparison instead of comparing with others, are suggested to be effective in enhancing students’ autonomous motivation.^34^ The study also provided support for TCM predictions using change scores. Results provide some support for the use of TCM as a basis for the development of interventions that promote PE students’ sport injury prevention in an out‐of‐school context. The current findings lay the groundwork for a broader test of TCM‐based interventions to prevention sport injury in representative sample of school students. It might be useful in to inform TCM‐based interventions in other educational settings, such as science and mathematics education,^22^, ^63^ physical education,^17^ and university education.^23^

## CONFLICT OF INTEREST

The authors declare that they have no conflict of interest.

## Data Availability

The data that support the findings of this study are available from the corresponding author upon reasonable request.

## Footnotes

^1^
Complete study measures and their detailed description (Appendix I) are available online at https://osf.io/gw3cu/.
